# Supplementary material for: Larval precompetency and settlement behaviour in 25 Indo-Pacific coral species
Source: Commun Biol. 2024 Jan 31;7:142. doi: 10.1038/s42003-024-05824-3 (PMC10830509; doi:10.1038/s42003-024-05824-3)
Supplement: Supplementary file 2 — Supplementary material [file 42003_2024_5824_MOESM2_ESM.pdf]

Supplementary Information for:
Larval precompetency and settlement behaviour in 25 Indo-Pacific coral species

Carly J Randall[1,2*], Christine Giuliano[1], Briony Stephenson[1], Taylor N Whitman[1,2], Cathie A Page[1], Eric A Treml[3], Murray Logan[1], Andrew P Negri[1]

[1]Australian Institute of Marine Science, Townsville QLD, Australia

[2]AIMS@JCU, Townsville QLD, Australia

[3]Australian Institute of Marine Science, Perth WA, Australia

*Correspondence to: c.randall@aims.gov.au

Data Availability Statement

The permanent data record and raw data can be found here:
https://apps.aims.gov.au/metadata/view/b5a63724-a339-488e-ae4d-b8a304371600

Code Availability Statement

Code is available at the github repository here:
https://github.com/open-AIMS/larval_settlement_competency

Supplementary Tables

Table S1. Table of known or estimated precompetency periods and maximum pelagic larval durations (PLD) for broadcast spawning scleractinian species.

Species	Precompetency period (d)	Maximum pelagic larval duration (PLD) (d)	Reference(s)
Acanthastrea lordhowensis	-	78	1
Acropora clathrata	5	-	2
Acropora cytherea	6	-	2
Acropora digitifera	3	-	3
Acropora gemmifera	3.8	34	4,5
Acropora humilis	2.6	-	5
Acropora hyacinthus	3	91+	6,7
Acropora latistella	-	209	8
Acropora longicyathus	4	-	7
Acropora lordhowensis	12	-	1
Acropora millepora	3.5; 4	60	4,5,9
Acropora nasuta	3	100+	3,8
Acropora spathulata	-	100+	8
Acropora tenuis / kenti	-	20 (modelled); 100+	10,11
Acropora valida	1.9; 4	110	4,5,9
Cyphastrea serailia	-	26	1
Dipsastraea (Favia) chinensis	1-2	56-63	12
Dipsastraea (Favia) favus	7	-	13
Dipsastraea (Favia) pallida	4.5	195	8,14
Fungia scutaria	5	-	15
Galaxea fascicularis	4.5		14
Goniastrea aspera	4.5; 2-3; 4.6	35; 63-70; 215	8,11,12,14,16
Goniastrea australensis	17	56	1,17
Goniastrea favulus	2.25; 6	-	14,18
Goniastrea retiformis	1.4; 5	36	4,5,14
Heliopora coerulea	-	30	19
Montastraea magnistellata	-	244	8
Montipora digitata	4	89+	14,20
Pectinia Paeonia	-	209	8
Platygyra daedalea	2.5, 3.1; 3	34; 105	4,5,18,21
Platygyra lamellina	8-9	-	13
Pocillopora damicornis	-	100, 103	10,19,22
Pseudodiploria strigosa	3	20	23

Table S2. Metadata for spawning, including the population, spawn date and spawn time for each species, and the number of broodstock colonies that contributed to each culture.

Species	Population	Spawn Date	Spawn Start Time	# Broodstock
Acropora austera	Davies and Backnumbers Reefs	28/11/2018	19:58	5
Acropora glauca	Keppel Islands	20/11/2019	21:10-21:40	7
Acropora hyacinthus	Keppel Islands	20/11/2019	22:07	7
Acropora intermedia	Keppel Islands	20/11/2019	20:57-21:50	4
Acropora longicyathus	Palm Islands	31/10/2018	19:05	6
Acropora loripes	Davies and Backnumbers Reefs	28/11/2018	19:40	6
Acropora micropthalma	Davies and Backnumbers Reefs	28/11/2018	20:26	3
Acropora millepora	Palm Islands	28/10/2018	20:40	7
Acropora muricata	Davies and Backnumbers Reefs	27/11/2018	21:16-21:40	5
Acropora cf. kenti (tenuis) [24]	Palm Islands	28/10/2018	18:25	15
Diploastrea heliopora	Davies and Backnumbers Reefs	27/11/2018	22:55-23:15	3+ ♂, 2 ♀
Dipsastraea (Favia) matthaii	Davies and Backnumbers Reefs	28/11/2018	21:20	3
	Davies and Backnumbers Reefs	27/11/2018	22:05	2+
Dipsastraea (Favia) pallida	Davies and Backnumbers Reefs	28/11/2018	23:35	3
Galaxea fascicularis	Palm Islands	17/11/2019	19:14-20:00	3 ♂, 3 ♀
Goniastrea retiformis	Palm Islands	29/10/2018	20:50	9
	Davies and Backnumbers Reefs	27/11/2018	20:35	5
	Palm Islands	16/11/2019	20:00 (est)	6
	Palm Islands	17/11/2019	20:00 (est)	not recorded
Lobophyllia corymbosa	Davies and Backnumbers Reefs	28/11/2018	19:00	2
	Davies and Backnumbers Reefs	29/11/2018	19:00 (est)	not recorded
Lobophyllia hemprichii	Davies and Backnumbers Reefs	28/11/2018	19:10	5
Montipora aequituberculata	Palm Islands	31/10/2018	20:00	4
	Keppel Islands	20/11/2019	19:45	6
Montipora digitata	Orpheus Island	4/04/2018	19:21	9
Mycedium elephantotus	Davies and Backnumbers Reefs	28/11/2018	21:06	4
Oulophyllia crispa	Palm Islands	20/11/2019	21:35	3
Pachyseris speciosa	Davies and Backnumbers Reefs	28/11/2018	19:00-20:50	1 ♂, 2 ♀
Platygyra daedalea	Palm Islands (13) + SeaSim experimental (originated from Palm Islands) (3)	29/10/2018	18:45	16
	SeaSim experimental (originated from Palm Islands)	28/11/2018	18:20	not recorded
Porites cylindrica	Palm Islands	28/10/2018	21:05	1+ ♂, 1 ♀
Porites lobata	Palm Islands	18/11/2019	21:40	2 ♂, 2 ♀

Supplementary Figures

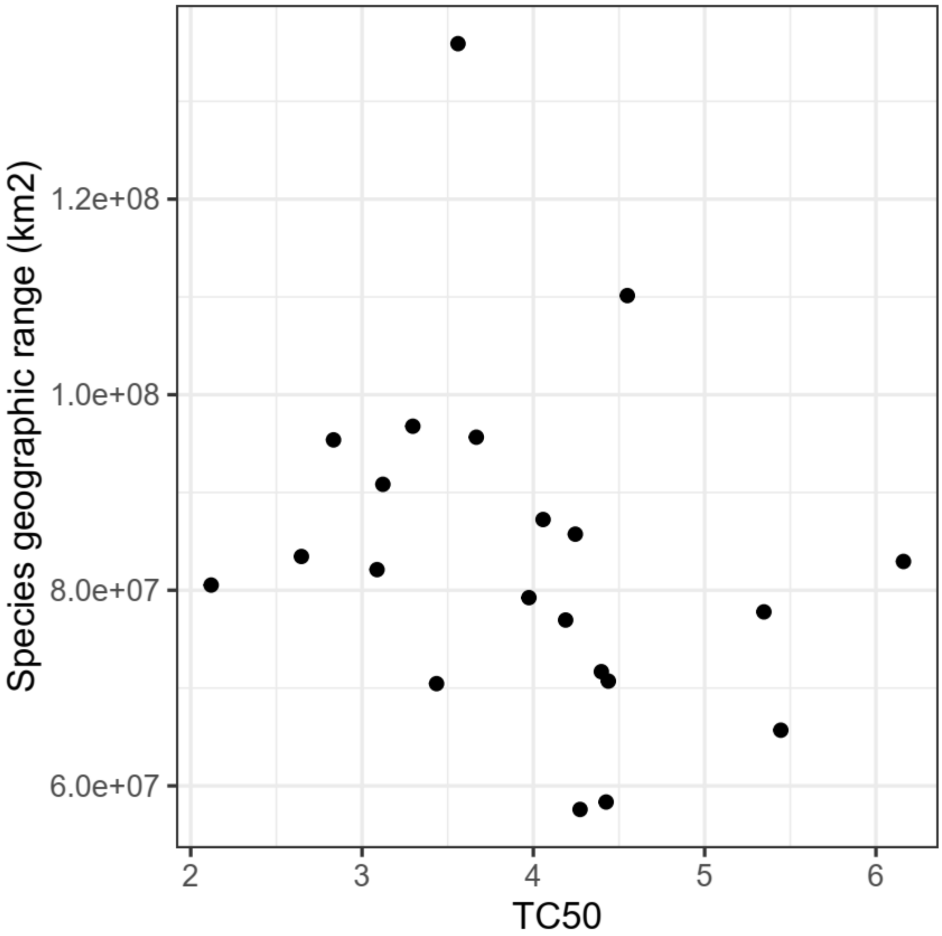

Figure S1. Modelled time to settlement competency (TC50) plotted against species geographic range estimates from [25]. No significant correlation was observed.

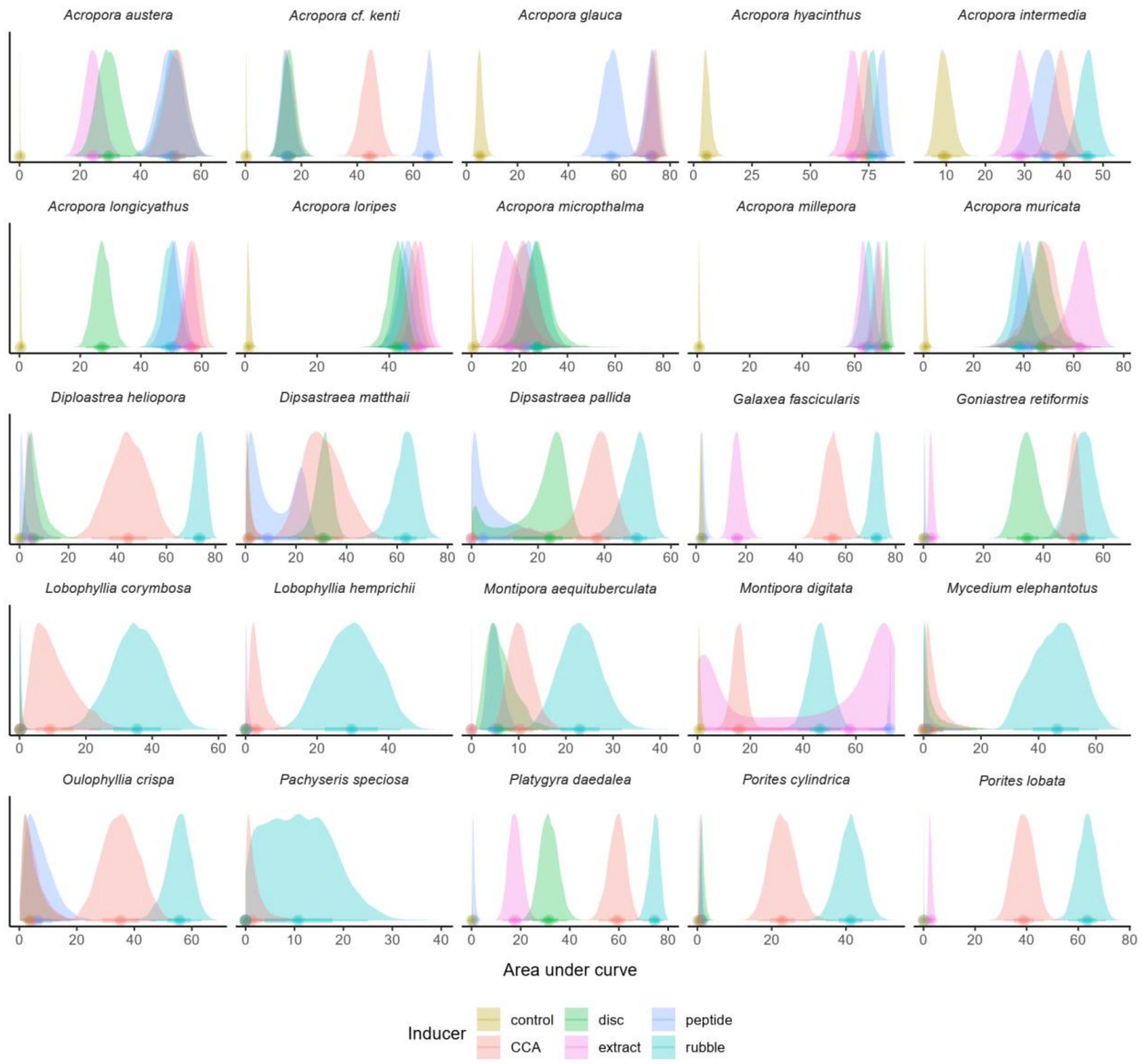

Figure S2. Posterior distributions of the areas under the curve, for each cue and species, from a Bayesian generalized additive hierarchical model and calculated by numerical integration under each spline (Figure 4). Generally, the larger the area, the more effective the cue at inducing settlement. Areas whose posterior distributions overlap are likely not significantly different from one another (see pairwise exceedance probabilities in Supplementary Code).

Supplementary References

1. Wilson, J. R. & Harrison, P. L. Settlement-competency periods of larvae of three species of scleractinian corals. *Marine Biology* **131**, 339–345 (1998).
2. Mohd Hanapiah, M. F. *et al.* Broadcast spawning pattern and pelagic larval duration of Acropora cytherea and A. Clathrata from inshore reef area in Kuantan coastal region. *Sci. herit. j.* **4**, 51–55 (2020).
3. Morse, Anc. *et al.* An ancient chemosensory mechanism brings new life to coral reefs. *The Biological Bulletin* **191**, 149–154 (1996).
4. Connolly, S. R. & Baird, A. H. Estimating dispersal potential for marine larvae: dynamic models applied to scleractinian corals. *Ecology* **91**, 3572–3583 (2010).
5. Figueiredo, J., Baird, A. H. & Connolly, S. R. Synthesizing larval competence dynamics and reef-scale retention reveals a high potential for self-recruitment in corals. *Ecology* **94**, 650–659 (2013).
6. Harrison, P. L. *et al.* Mass spawning in tropical reef corals. *Science* **223**, 1186–1189 (1984).
7. Harrison, P. L. Settlement competency periods and dispersal potential of scleractinian reef coral larvae. in *Proceedings of the 10th International Coral Reef Symposium* 78–82 (2006).
8. Graham, E. M., Baird, A. H. & Connolly, S. R. Survival dynamics of scleractinian coral larvae and implications for dispersal. *Coral Reefs* **27**, 529–539 (2008).
9. Ayre, D. J. & Hughes, T. P. Genotypic diversity and gene flow in brooding and spawning corals along the Great Barrier Reef, Australia. *Evolution* **54**, 1590–1605 (2000).
10. Richmond, R. H. Competency and dispersal potential of planula larvae of a spawning versus brooding coral. in *Proceedings of the 6th International Coral Reef Symposium* vol. 2 827–831 (1988).
11. Graham, E. M., Baird, A. H., Connolly, S. R., Sewell, M. A. & Willis, B. L. Rapid declines in metabolism explain extended coral larval longevity. *Coral Reefs* **32**, 539–549 (2013).
12. Nozawa, Y. & Harrison, P. L. Temporal settlement patterns of larvae of the broadcast spawning reef coral Favites chinensis and the broadcast spawning and brooding reef coral Goniastrea aspera from Okinawa, Japan. *Coral Reefs* **24**, 274–282 (2005).
13. Shlesinger, Y. & Loya, Y. Larval development and survivorship in the corals *Favia favus* and *Platygyra lamellina*. in *Coelenterate Biology: Recent Research on Cnidaria and Ctenophora* (eds. Williams, R. B., Cornelius, P. F. S., Hughes, R. G. & Robson, E. A.) 101–108 (Springer Netherlands, 1991). doi:10.1007/978-94-011-3240-4_14.
14. Babcock, R. C. & Heyward, A. J. Larval development of certain gamete-spawning scleractinian corals. *Coral Reefs* **5**, 111–116 (1986).

15. Schwarz, J. A., Krupp, D. A. & Weis, V. M. Late larval development and onset of symbiosis in the scleractinian coral *Furgia scutaria*. *Biological Bulletin* **196**, 70–79 (1999).

16. Doropoulos, C., Gómez-Lemos, L. A. & Babcock, R. C. Exploring variable patterns of density-dependent larval settlement among corals with distinct and shared functional traits. *Coral Reefs* **37**, 25–29 (2018).

17. Kojis, B. L. & Quinn, N. J. Aspects of sexual reproduction and larval development in the shallow water hermatypic coral, *Goniastrea australensis* (Edwards and Haime, 1857). *Bulletin of Marine Science* **31**, 558–573 (1981).

18. Miller, K. & Mundy, C. Rapid settlement in broadcast spawning corals: implications for larval dispersal. *Coral Reefs* **22**, 99–106 (2003).

19. Harii, S., Kayanne, H., Takigawa, H., Hayashibara, T. & Yamamoto, M. Larval survivorship, competency periods and settlement of two brooding corals, Heliopora coerulea and Pocillopora damicornis. *Marine Biology* **141**, 39–46 (2002).

20. Randall, C. J., Giuliano, C., Mead, D., Heyward, A. J. & Negri, A. P. Immobilisation of living coral embryos and larvae. *Sci Rep* **9**, 14596 (2019).

21. Nozawa, Y. & Harrison, P. L. Larval settlement patterns, dispersal potential, and the effect of temperature on settlement of larvae of the reef coral, *Platygyra daedalea*, from the Great Barrier Reef. in *Proceedings of the 9th International Coral Reef Symposium* vol. 1 409–416 (2000).

22. Richmond, R. Energetics, competency, and long-distance dispersal of planula larvae of the coral *Pocillopora damicornis*. *Marine Biology* **93**, 527–533 (1987).

23. Davies, S. W., Strader, M. E., Kool, J. T., Kenkel, C. D. & Matz, M. V. Modeled differences of coral life-history traits influence the refugium potential of a remote Caribbean reef. *Coral Reefs* **36**, 913–925 (2017).

24. Bridge, T. C. L. *et al.* A *tenuis* relationship: traditional taxonomy obscures systematics and biogeography of the ‘ *Acropora tenuis* ’ (Scleractinia: Acroporidae) species complex. *Zoological Journal of the Linnean Society* zlad062 (2023) doi:10.1093/zoolinnean/zlad062.

25. Hughes, T. P., Connolly, S. R. & Keith, S. A. Geographic ranges of reef corals (Cnidaria: Anthozoa: Scleractinia) in the Indo-Pacific: *Ecological Archives* E094-150. *Ecology* **94**, 1659 (2013).
