# Supplementary material for: Larval precompetency and settlement behaviour in 25 Indo-Pacific coral species
Source: Commun Biol. 2024 Jan 31;7:142. doi: 10.1038/s42003-024-05824-3 (PMC10830509; doi:10.1038/s42003-024-05824-3)
Supplement: Supplementary file 3 — Description of Supplementary Materials [file 42003_2024_5824_MOESM3_ESM.docx]

**Description of Additional Supplementary Files**

**File name:** Supplementary Data 1

**Description:** Percentage settlement (mean ± SE) by coral species, larval age (d) and

experimental treatment. CCA = crustose coralline algae Porolithon cf. onkodes. Treatments

are as described in the Methods. Species abbreviations as in Table 1
