# Supplementary material for: Larval precompetency and settlement behaviour in 25 Indo-Pacific coral species
Source: Commun Biol. 2024 Jan 31;7:142. doi: 10.1038/s42003-024-05824-3 (PMC10830509; doi:10.1038/s42003-024-05824-3)
Supplement: Supplementary file 5 — Reporting summary [file 42003_2024_5824_MOESM5_ESM.pdf]

nature portfolio

Corresponding author(s): Carly J Randall

Last updated by author(s): Jan 2, 2024

Reporting Summary

Nature Portfolio wishes to improve the reproducibility of the work that we publish. This form provides structure for consistency and transparency in reporting. For further information on Nature Portfolio policies, see our Editorial Policies and the Editorial Policy Checklist.

Statistics

For all statistical analyses, confirm that the following items are present in the figure legend, table legend, main text, or Methods section.

n/a	Confirmed	
☐	☒	The exact sample size (*n*) for each experimental group/condition, given as a discrete number and unit of measurement
☐	☒	A statement on whether measurements were taken from distinct samples or whether the same sample was measured repeatedly
☐	☒	The statistical test(s) used AND whether they are one- or two-sided<br>*Only common tests should be described solely by name; describe more complex techniques in the Methods section.*
☒	☐	A description of all covariates tested
☐	☒	A description of any assumptions or corrections, such as tests of normality and adjustment for multiple comparisons
☐	☒	A full description of the statistical parameters including central tendency (e.g. means) or other basic estimates (e.g. regression coefficient) AND variation (e.g. standard deviation) or associated estimates of uncertainty (e.g. confidence intervals)
☐	☒	For null hypothesis testing, the test statistic (e.g. *F*, *t*, *r*) with confidence intervals, effect sizes, degrees of freedom and *P* value noted<br>*Give P values as exact values whenever suitable.*
☐	☒	For Bayesian analysis, information on the choice of priors and Markov chain Monte Carlo settings
☐	☒	For hierarchical and complex designs, identification of the appropriate level for tests and full reporting of outcomes
☐	☒	Estimates of effect sizes (e.g. Cohen's *d*, Pearson's *r*), indicating how they were calculated

Our web collection on statistics for biologists contains articles on many of the points above.

Software and code

Policy information about availability of computer code

Data collection	No software was used to collect data
Data analysis	All data anlyses were conducted in the open source program R and all statistical packages and libraries are listed in the methods section. No custom code was developed. All data, code and outputs will be freely available at a permanent data repository link upon publication.

For manuscripts utilizing custom algorithms or software that are central to the research but not yet described in published literature, software must be made available to editors and reviewers. We strongly encourage code deposition in a community repository (e.g. GitHub). See the Nature Portfolio guidelines for submitting code & software for further information.

Data

Policy information about availability of data

All manuscripts must include a data availability statement. This statement should provide the following information, where applicable:

- Accession codes, unique identifiers, or web links for publicly available datasets
- A description of any restrictions on data availability
- For clinical datasets or third party data, please ensure that the statement adheres to our policy

All data and analysis code will be freely available at a permanent data repository link, hosted by the Australian Institute of Marine Science, upon publication.

Research involving human participants, their data, or biological material

Policy information about studies with human participants or human data. See also policy information about sex, gender (identity/presentation), and sexual orientation and race, ethnicity and racism.

Reporting on sex and gender	N/A
Reporting on race, ethnicity, or other socially relevant groupings	N/A
Population characteristics	N/A
Recruitment	N/A
Ethics oversight	N/A

Note that full information on the approval of the study protocol must also be provided in the manuscript.

Field-specific reporting

Please select the one below that is the best fit for your research. If you are not sure, read the appropriate sections before making your selection.

☐ Life sciences ☐ Behavioural & social sciences ☒ Ecological, evolutionary & environmental sciences

For a reference copy of the document with all sections, see nature.com/documents/nr-reporting-summary-flat.pdf

Ecological, evolutionary & environmental sciences study design

All studies must disclose on these points even when the disclosure is negative.

Study description	We performed replicated 24-hr settlement assays starting during precompetency and tracked larval settlement up to 77 days post-spawning, to (i) define the precompetency period, (ii) test settlement responses to a variety of common cues, and (iii) improve our predictions of settlement competency windows. We tested up to 5 experimental treatments and a negative control. These included: CCA, biofilm disc, neuropeptide, CCA extract, and live reef rubble. There were typically 10 larvae per replicate well and 6 replicate wells per treatment per time and the number of timepoints tested varied by species.
Research sample	The research samples consisted of batches of coral larvae (10 larvae per replicate well), generated from spawning broodstock collected from the reef. These larvae were generated from 25 species of Scleractinian corals, which are defined with their taxonomy in the manuscript. The samples are meant to represent the central mid-shelf Great Barrier Reef coral populations and the sites of collection, as well as the numbers of broodstock that contributed to each larval culture are described in a table.
Sampling strategy	No sample-size calculation was performed a priori. Instead, industry standard larval densities (1 per ml, in 10 ml wells) and replicate sample sizes (6 replicate wells per treatment) were applied. This sample size was sufficient to detect differences in larval settlement preference amongst settlement cues.
Data collection	All assays were scored 24 to 36 hours after set-up by counting the number of larvae that had and had not demonstrated settlement under a standard dissection microscope. In species with fluorescent larvae, identification was aided by applying a 440-460 nm fluorescent LED lighting system with a 500 nm longpass emission filter (NIGHTSEA® Royal Blue) to the dissection microscope. Data were collected by all observers using a standard data sheet, at the microscope at the time of observation, by hand. All observers were trained in the identification of settled spat and any difficult identifications were confirmed by a second observer. Data were then entered into excel by each observer and a second observer performed a quality assurance and quality control data check of the entered data using the hand-recorded data sheets.
Timing and spatial scale	The timing of spawning, the start and end dates of all assays, and and the timing and frequency of sampling for every species and year can be found in the raw data that will be freely available upon publication. The temporal scale of assessment varied by species and depended on larval availability, and the cohort testing was dictated by seasonal mass coral spawning. All taxa that were included in time-series assessments met minimum requirements for time of testing post-spawning and frequency of sampling for modelling time to competency. Cue comparison analyses could be completed on all taxa and the timing of all assays is recorded in the available data.
Data exclusions	Taxa were excluded from the time to competency analysis if they had not been sampled within 4 days of spawning.
Reproducibility	Temporal replication was used to validate the responses of the larvae to each of the cues tested. No attempts were made to reproduce the experiment over multiple cohorts.
Randomization	Treatment placement within well plates was done using a formal randomization process, in groups of 3. This randomization allowed us to remove any bias associated with placement of the well plate on the lab bench, but limited the likelihood of mistakes arising

from loading fully randomized wells. Larvae were sampled haphazardly from the culture tank and loaded into the wells in the randomized order.

Blinding

Blinding was not possible with this study because the settlement cues are visually identifiable (i.e. CCA is discernable from reef rubble which is distinct from a biofilm disc) and the larval species can typically be identified, at least to genus-level.

Did the study involve field work? ☐ Yes ☒ No

Reporting for specific materials, systems and methods

We require information from authors about some types of materials, experimental systems and methods used in many studies. Here, indicate whether each material, system or method listed is relevant to your study. If you are not sure if a list item applies to your research, read the appropriate section before selecting a response.

Materials & experimental systems

n/a	Involved in the study
☒	☐ Antibodies
☒	☐ Eukaryotic cell lines
☒	☐ Palaeontology and archaeology
☐	☒ Animals and other organisms
☒	☐ Clinical data
☒	☐ Dual use research of concern
☒	☐ Plants

Methods

n/a	Involved in the study
☒	☐ ChIP-seq
☒	☐ Flow cytometry
☒	☐ MRI-based neuroimaging

Animals and other research organisms

Policy information about studies involving animals; ARRIVE guidelines recommended for reporting animal research, and Sex and Gender in Research

Laboratory animals

For laboratory animals, report species, strain and age OR state that the study did not involve laboratory animals.

Wild animals

Fecund coral colonies of 25 taxa were collected from locations along the Great Barrier Reef prior to the predicted 2017, 2018 and 2019 mass coral-spawning events in October and November each year Table S 2). All coral species names and collection locations are reported in the text. Corals were collected under permits G12/35236.1 and G19/43024.1 issued by the Great Barrier Reef Marine Park Authority (GBRMPA) via hammer and chisel, or from pre-established nursery racks. Once spawning was completed in the laboratory, and within 4 weeks, the colonies were returned to the site of collection and placed back on nursery racks.

Reporting on sex

No sex-based analyses were performed. Most taxa are hermaphroditic. When gonochoric, the numbers of males and females contributing to each culture are stated. Larvae were not sexed.

Field-collected samples

Please see wild animals above. Corals were transported to the National Sea Simulator (SeaSim) at the Australian Institute of Marine Science (AIMS) via ship and maintained in flow-through outdoor aquaria at ambient light and temperature conditions matching source locations.

Ethics oversight

No ethical approval was required as the animals are Cnidarians. All required permits were obtained from the Great Barrier Marine Park Authority.

Note that full information on the approval of the study protocol must also be provided in the manuscript.

Plants

Seed stocks

Report on the source of all seed stocks or other plant material used. If applicable, state the seed stock centre and catalogue number. If plant specimens were collected from the field, describe the collection location, date and sampling procedures.

Novel plant genotypes

Describe the methods by which all novel plant genotypes were produced. This includes those generated by transgenic approaches, gene editing, chemical/radiation-based mutagenesis and hybridization. For transgenic lines, describe the transformation method, the number of independent lines analyzed and the generation upon which experiments were performed. For gene-edited lines, describe the editor used, the endogenous sequence targeted for editing, the targeting guide RNA sequence (if applicable) and how the editor was applied.

Authentication

Describe any authentication procedures for each seed stock used or novel genotype generated. Describe any experiments used to assess the effect of a mutation and, where applicable, how potential secondary effects (e.g. second site T-DNA insertions, mosiacism, off-target gene editing) were examined.
